# Supplementary material for: Causal Relationship between Aging and Anorexia Nervosa: A White-Matter-Microstructure-Mediated Mendelian Randomization Analysis
Source: Biomedicines. 2024 Aug 16;12(8):1874. doi: 10.3390/biomedicines12081874 (PMC11351342; doi:10.3390/biomedicines12081874)
Supplement: Supplementary file 1 [file biomedicines-12-01874-s001.zip › Supplementary Figure/Figure S8/Leaveoneout plot of MR analysis for TL to ubm-b-1464.pdf]

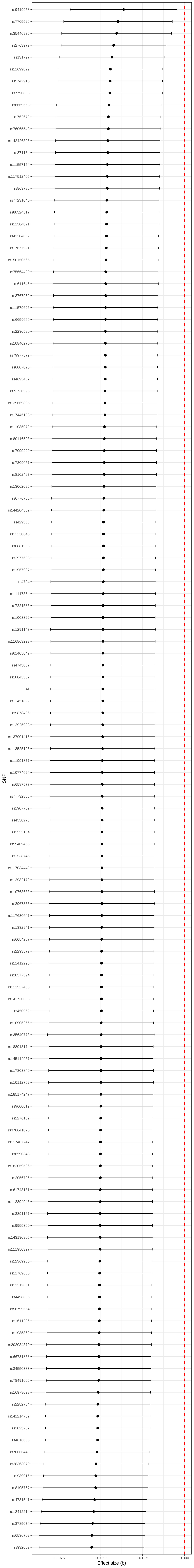

rs9419958
rs7705526
rs35446936
rs2763979
rs131797
rs11699829
rs5742915
rs7790856
rs6669563
rs762679
rs76065543
rs142426306
rs871134
rs11557154
rs117512405
rs869785
rs77231040
rs80324517
rs11584821
rs41304832
rs17677991
rs150150565
rs75664430
rs611646
rs3767952
rs11579626
rs6659669
rs2230590
rs10840270
rs79977579
rs6007020
rs4695407
rs73730598
rs139669835
rs17445108
rs11085072
rs80116508
rs7099229
rs7209057
rs8102497
rs13062095
rs6776756
rs144204502
rs429358
rs13230646
rs6881568
rs2977608
rs1957937
rs4724
rs11117354
rs7221585
rs1003322
rs1291143
rs116863223
rs61405042
rs4743037
rs10845387
A8
rs12451892
rs9878436
rs12925933
rs137901416
rs113525195
rs11991877
rs10774624
rs6587577
rs77732866
rs1907702
rs4530278
rs2555104
rs59409453
rs2538745
rs117034449
rs12932179
rs10768683
rs2967355
rs117630647
rs1332941
rs6054257
rs2293579
rs11412296
rs28577594
rs111527438
rs142730696
rs450962
rs10905255
rs35640778
rs188918174
rs145114957
rs17803849
rs10112752
rs185174247
rs9600019
rs2276182
rs376641875
rs117407747
rs6590343
rs182059586
rs2056726
rs61748181
rs112394943
rs3891167
rs9955360
rs143190905
rs111950327
rs12369950
rs11769630
rs11212631
rs4498805
rs56799554
rs1611236
rs1985369
rs202034370
rs66731853
rs34550383
rs78491606
rs16978028
rs2282764
rs141214782
rs1023767
rs4616688
rs76666449
rs28363070
rs939916
rs8105767
rs4731541
rs12412214
rs3785074
rs6536702
rs932002
SNP
−0.075
−0.050
−0.025
0.000
Effect size (b)
